# The impact of tumor immunogenicity on cancer pain phenotype using syngeneic oral cancer mouse models

**DOI:** 10.3389/fpain.2022.991725

**Published:** 2022-09-12

**Authors:** Nicole L. Horan, Lisa A. McIlvried, Megan A. Atherton, Mona M. Yuan, John C. Dolan, Nicole N. Scheff

**Affiliations:** ^1^Department of Neurobiology, University of Pittsburgh, Pittsburgh, PA, United States; ^2^Hillman Cancer Center, University of Pittsburgh Medical Center, Pittsburgh, PA, United States; ^3^College of Dentistry, New York University, New York, NY, United States

**Keywords:** nociception, oral cancer, immunogenicity, mouse model, syngeneic

## Abstract

Head and neck squamous cell carcinoma (HNSCC) patients report severe function-induced pain at the site of the primary tumor. The current hypothesis is that oral cancer pain is initiated and maintained in the cancer microenvironment due to secretion of algogenic mediators from tumor cells and surrounding immune cells that sensitize the primary sensory neurons innervating the tumor. Immunogenicity, which is the ability to induce an adaptive immune response, has been widely studied using cancer cell transplantation experiments. However, oral cancer pain studies have primarily used xenograft transplant models in which human-derived tumor cells are inoculated in an athymic mouse lacking an adaptive immune response; the role of inflammation in oral cancer-induced nociception is still unknown. Using syngeneic oral cancer mouse models, we investigated the impact of tumor cell immunogenicity and growth on orofacial nociceptive behavior and oral cancer-induced sensory neuron plasticity. We found that an aggressive, weakly immunogenic mouse oral cancer cell line, MOC2, induced rapid orofacial nociceptive behavior in both male and female C57Bl/6 mice. Additionally, MOC2 tumor growth invoked a substantial injury response in the trigeminal ganglia as defined by a significant upregulation of injury response marker ATF3 in tongue-innervating trigeminal neurons. In contrast, using a highly immunogenic mouse oral cancer cell line, MOC1, we found a much slower onset of orofacial nociceptive behavior in female C57Bl/6 mice only as well as sex-specific differences in the tumor-associated immune landscape and gene regulation in tongue innervating sensory neurons. Together, these data suggest that cancer-induced nociceptive behavior and sensory neuron plasticity can greatly depend on the immunogenic phenotype of the cancer cell line and the associated immune response.

## Introduction

Head and neck squamous cell carcinoma (HNSCC) is the sixth most common cancer worldwide with 890,000 new cases a year and a 5-year survival rate of 50% (1–3). Among all cancers, HNSCC is one of the most painful (4–6); the dense oral innervation of the trigeminal nerve (7) may additionally explain the localization of pain in HNSCC which is not seen in other painful cancers (i.e., gastrointestinal, pelvic). Oral cancer pain is further aggravated by stimulations of the oral cavity including deglutition, mastication, and speech. The direct association between oral cancer and pain has been proven through a reduction of associated pain through the surgical excision of the tumor (8). Additionally, studies have shown that nodal metastasis is associated with more frequent and intense oral-cancer-associated pain, indicating a positive correlation between disease progression and pain intensity (4).

Although clinical data demonstrates the association between oral cancer and pain, preclinical oral cancer pain models to understand the underlying pain neurobiology are currently lacking. HNSCC pain has been primarily characterized either in athymic nude mice lacking a complete adaptive immune response or in chronic carcinogen-induced cancer pain mouse models using 4-nitroquinoline-1-oxide (4NQO) (9). Both models have characteristics that limit their inherent translatability and investigative potential for pharmacologic interventions. The athymic models fail to incorporate the role of the adaptive immune response which is critical in determining the nature and potential function of the inflammatory infiltrate in cancer-associated pain as well-tumor progression. The 4NQO model, which can be executed in an immunocompetent mouse, is associated with characteristic histopathology of papilloma which can occur anywhere in the oral cavity; however, papillary lesions are rarely seen in human oral cancer (10) and there is a lack of consensus regarding the role of papilloma in cancer progression within the 4NQO model (11). Lastly, there is further debate in the literature regarding tumor size and its impact on nociception. Several studies report no correlation between tumor size and patient-reported pain or nociceptive behavior within a mouse xenograft model (12, 13). Other studies report an association between tumor size and patient-reported pain as well as associated nociceptive behavior in a carcinogen mouse model (14).

The ability to assess the neuroimmune interactions associated with oral cancer pain may be critical to the inherent translatability of preclinical cancer pain research. Previous studies have demonstrated that tumor cells and immune cells have distinct roles in cancer-related pain (15, 16). Clinically, HNSCC shows deficiencies in the type 1 interferon (IFN-I) induction pathway that allows for evasion of innate immune detection (17). However, there is an inherent variability among cancer cells regarding their immunogenicity such that some HNSCC cancers interact with the immune system and are sensitive to immunotherapeutic treatments (e.g., immune checkpoint inhibitors) while other HNSCC cancers can completely evade immune interaction (18). While cancer itself has the potential to evade immune detection, the natural inflammatory response to cancer may impact the subsequent growth rate of the tumor as well as the onset and progression of cancer-induced nociceptive behavior. Emerging syngeneic transplant mouse models offer a holistic alternative to study cancer-neuro-immune interactions. These models use mouse oral tumor cells and can offer reproducibility and neuroimmune evaluation; however, no nociceptive characterization of these syngeneic oral cancer models has been reported to date. To better understand the immune system's role in oral cancer-related pain, we assessed immunogenicity of four mouse oral cancer cell lines and characterized the nociceptive phenotypes of two syngeneic orthotopic transplant mouse oral cancer models by quantifying nociceptive behavior, cancer-induced immune infiltrate, and changes in pain-related genes and proteins within the peripheral sensory nervous system in tumor-bearing animals compared to time-matched sham mice.

## Methods

### Cell culture

All cell lines were cultured in 10 cm diameter cell culture dishes at 37°C with 5% CO2. Mouse oral squamous cell carcinoma lines, MOC1, MOC2, MOC22 (Kerafast), and a 4-Nitroquinoline 1-oxide (4NQO) derived NOOC1 cell line were all cultured in IMDM/F12 (2:1; Thermo Fisher Scientific) supplemented with 5% fetal bovine serum (Thermo Fisher Scientific), penicillin streptomycin solution (Penn/Strep, Thermo Fisher Scientific), 5 μg/mL insulin (Sigma-Aldrich), 40 ng/mL hydrocortisone (Sigma-Aldrich), and 5 ng/mL epidermal growth factor (EMD Millipore). Cell pellets for gene expression analysis from all cell lines were collected from a passage numbers <14, with a total of 3 different passages utilized to assess cancer cell immunogenicity. Cells were grown to 50% confluency and then treated with either vehicle [0.1% albumin bovine serum (BSA)] or 100 ng/ml interferon gamma (IFN-γ, stock solution 100 μg/mL in 1 mM BSA; R&D Systems) for 24 h. Cells were then harvested by 0.25% trypsin-EDTA treatment, pelleted (400 xg for 4 min), snap frozen, and stored at −80°C until needed.

### Animals

Adult (6–12 weeks, 20–30 g) male and female C57BL/6 (stock #000664; Jackson Labs, Bar Harbor, ME) mice were used for all experiments. All mice were housed in a temperature-controlled room on a 12:12-h light cycle (0,700–1,900 h light), with unrestricted access to food and water. Researchers were trained under the Animal Welfare Assurance Program. All procedures were approved by the University of Pittsburgh Institutional Animal Care and Use Committee and performed in accordance with the National Institutes of Health guidelines for the use of laboratory animals in research.

### Retrograde labeling of tongue primary afferent neurons

The retrograde tracer 1,10-dioctadecyl-3,3,30,30-tetramethy lindocarbocyanine perchlorate (DiI, Invitrogen, Carlsbad, CA, USA) was injected peripherally into adult C57Bl/5 mice under 3–5% isoflurane anesthesia (Covetrus) within the anterior lateral tongue to retrogradely label tongue afferents. The tracer was dissolved at 170 mg/mL in DMSO (Thermo Fisher Scientific), diluted 1:10 in 0.9% sterile saline (Thermo Fisher Scientific) and injected bilaterally using a 30 g needle for a total volume of 5–7 μL per tongue.

### Syngeneic orthotopic oral cancer mouse model

To generate the syngeneic orthotopic oral cancer mouse model, adult male and female mice under 3–5% isoflurane anesthesia were inoculated into the anterior lateral portion of the tongue with either 7.5 × 10^5^ MOC1 cells or 2 × 10^4^ MOC2 in 30 μL of a mixture of DMEM (Life Technologies) and Matrigel (Corning) at a 1:1 ratio as previously described (19). Injection of DMEM and Matrigel alone was used as control (i.e., sham). No direct statistical comparison was made between MO*C1*- and MO*C2*-tumor bearing mice as data had to be collected at significantly different time points due to differential rate of tumor development. Harvest timepoints were set at *post-*inoculation *day* 40 (PID 40) or post inoculation *day* 14 (PID 14) for MO*C1* and MO*C2, respectively*, with the exception of tissue harvest for cytometric evaluation (See *Analytical Flow Cytometry*). Weight was recorded weekly starting on the day of inoculation. Tumor volume was quantified every 3–4 days using calipers and calculated using the volume of ellipsoid formula V = 4/3π × Length × Width × Height. On the respective harvest day, mice under 3–5% isoflurane anesthesia were transcardially perfused with PBS; for histological studies tissue was subsequently perfused with 4% paraformaldehyde (PFA; Thermo Fisher Scientific). Trigeminal ganglia (TG) tissue was harvested, snap-frozen, and stored at −80°C until needed for real time quantitative PCR or fixed in 4% PFA for 1 h and stored in 30% sucrose until needed for immunohistochemistry. Tongue tissue as well as submandibular and cervical lymph nodes were harvested and immediately processed for cytometric evaluation of immune cells. Mice were excluded from the study if (1) no tumor developed at the time of harvest, and (2) if the mouse bit through the tongue tumor resulting in an ulcer or loss of tongue tissue at the time of harvest.

### Nociceptive behavior

The dolognawmeter assay and automated device quantifies gnawing activity. The outcome variable (gnaw-time) is the time required by a rodent to gnaw through the second of two obstructing dowels in series (a discrete gnawing task) that block escape of a rodent confined in a narrow tube. Extended gnaw-time relative to baseline values is a validated index of orofacial nociception in mice with oral cancer (20). Each mouse is placed into a confinement tube, where forward movement of the rodent in the tube is obstructed by 2 polymer dowels. The mouse voluntarily gnaws through both dowels to escape the device. A digital timer automatically records the duration required for the mouse to sever the second dowel. To acclimatize the mice and improve consistency in gnawing behavior, all mice were trained for 7–9 sessions in the dolognawmeter. Training was accomplished by placing the mice in the device and allowing them to gnaw through the obstructing dowels in the same manner as the subsequent experimental gnawing trials. For the oral cancer pain model, a baseline gnaw-time (mean of the final 3 training sessions) was established for each mouse followed by behavioral testing twice per week for up to 5 weeks. Each mouse was compared to its own baseline gnaw-time, and data are presented as a percent change ± standard error of the mean.

### Quantitative real-time PCR

Total RNA was isolated from pelleted cells (1–1.5 × 10^6^) from cell lines and whole trigeminal ganglia (TG) using the Qiagen RNeasy Plus Mini Kit (Qiagen Inc.). Reverse transcription was performed with Quantitect Reverse Transcription Kit (Qiagen Inc.) according to the manufacturer's instructions. For single cell analysis, DiI-positive single neurons were dissociated as previously described (19, 21, 22), identified using fluorescence microscopy, and limited to ≤ 25 μm in diameter. Cells were then picked up using glass capillaries (World Precision Instruments) which were held by micromanipulator (Sutter Instruments), headstage (EPC10 HEKA) and electrode holder under brightfield optics. Each cell was transferred into a 0.2 ml PCR tube containing 9 μl of single cell lysis solution and DNase I from Single Cell-to-CT^TM^ Kit (Thermo Fisher Scientific), incubated for 2 min and immediately stored at −80°C until further use. Cells were collected within 1 h of removal from the incubator and within 8 h of removal from the animals (*n* = 2 C57Bl/6 mice). Reverse transcription, cDNA pre-amplification and Real-Time PCR were executed per manufacturer's instructions. Relative expression levels of pain-related genes were assessed using TaqMan Gene Expression Assays and TaqMan Gene Expression Master Mix (Thermo Fisher Scientific), using a 96 well-Quantstudio 3 Real-Time PCR System (Thermo Fisher Scientific). Assays from Life Technologies were used to probe for the following genes: *Atf3* (Mm00476032_m1), *Trpv1* (Mm01246302_m1), *Calca* (Mm00801463_g1). The housekeeping genes *Actb* (Mm02619580_g1), *Gapdh* (Mm99999915_g1), and *Gusb* (Mm01197698_m1) were used as the internal control genes. All samples were run in duplicate or triplicate to account for pipetting error. Relative fold change of gene expression data in cancer mice compared to sham mice was calculated using the 2^−ΔΔCt^ method. For single cell PCR, any cell with a *Gapdh* cycle threshold (Ct) of 25 or higher was excluded from further analysis. Genes were considered “not expressed” if one sample either failed to detect expression or the Ct was above 35.

### Immunohistochemistry

At least 10 days prior to tissue harvest or cancer/sham inoculation, the retrograde tracer DiI was injected peripherally into the anterior lateral tongue to retrograde label tongue afferents as described above. Mice under 3–5% isoflurane anesthesia were transcardially perfused with PBS followed by 4% PFA. The TG were dissected, postfixed for 1 h in 4% PFA, and cryoprotected in 30% sucrose at 4°C overnight. TG were then embedded in Tissue-Tek OCT compound (Thermo Fisher Scientific), sectioned (14 μm), and mounted on Superfrost Plus slides (Thermo Fisher Scientific). Slides were then incubated in blocking solution comprised of PBS supplemented with Mg^2+^ and Ca^2+^ (PBS^+/+^, Life Technologies), 10% normal goat serum (Thermo Fisher Scientific), 0.01% Triton X (Sigma), 1% bovine serum albumin (Thermo Fisher Scientific), and 0.1% Tween-20 (Sigma) for 1 h at room temperature (RT). Slides were subsequently incubated overnight in PBS^+/+^ containing 1% bovine serum albumin and 0.1% Tween-20 and one of the following primary antibodies: rabbit anti-CGRP (1:500 at 4°C, Cell Signaling CAT#14959S), rabbit anti-TRPV1 (1:500 at RT, Alomone Labs Cat# ACC-030) or rabbit anti-ATF3 (1:250 at 4°C, Abcam Cat# ab207434). Following primary antibody incubation, slides were extensively washed in PBS^+/+^ and incubated in goat anti-rabbit Alexa fluor 488 (1:250, Jackson ImmunoResearch Cat#111-545-006) for 2 h at RT and cover-slipped with Fluoro-Gel II mounting media containing DAPI (Thermo Fisher Scientific). Using a Keyence BZ-X810 microscope with Keyence Imaging software, TG sections were photographed at 20x magnification within the intersection of the mandibular and maxillary branch, where most retrograde labeled trigeminal tongue neurons reside. Trigeminal ganglion neurons with distinct nuclei and at least 50% of the cell area labeled with DiI were counted in every fifth section (9 sections/mouse/treatment group). Nuclei were identified using nuclear stain, DAPI, in the mounting media as *well* as anatomically using a phase contrast brightfield image. To account for biological variance, *3–4* mice per group with an average of 154.4 ± 26.5 neurons per animal were quantified by blinded evaluators (NLH, MMY); NNS held the allocation keys. ImageJ software (NIH, Bethesda, MD) was used to count retrograde labeled neurons that overlapped with protein-specific immunoreactivity per animal.

### Analytical flow cytometry

Analytical cytometry was used to assess immune infiltrate in mouse tumors and bilateral submandibular and cervical (draining) lymph nodes from dissociated sham and MOC1- or MOC2-tumor tongue tissue. Mouse tongues were harvested and dissociated as previously described (23). Briefly, tongue tissue was dissected and minced in DMEM with pen/strep (Thermo Fisher Scientific), collagenase-H (0.5 mg/mL; Sigma-Aldrich, 34 units/mg), DNase (0.5 mg/mL) and 20 mM 4-(2-hydroxyethyl)-1-piperazine ethanesulfonic acid (HEPES, Thermo Fisher Scientific), and then incubated at 37°C for 1 h. The tissue was then mechanically dissociated using frosted glass slides, washed twice with fresh DMEM containing penn/strep and HEPES, resuspended in CaCl_2_/MgCl_2_ free phosphate buffered saline (Sigma-Aldrich) containing 3% FBS, 1 mM EDTA (Sigma-Aldrich) and filtered through a 40 μm cell strainer (Falcon brand, Thermo Fisher Scientific). To isolate subpopulations, cells were stained with the following fluorescently conjugated rat anti-mouse monoclonal antibodies: CD45-BUV 395 (BDbiosciences); CD3-BUV510 (Biolegend); CD11b-APC (Biolegend); CD8-PerCP Cy5.5 (Biolegend); CD11c-BV650 (Biolegend); Ly6g-BV711 (Biolegend); CD4-BUV37 (BDbiosciences); CD19-PE Dazzle (Biolegend); NK1.1-AF488 (Biolegend); MHCII-AF700 (Biolegend); Ly6c-BV421 (Biolegend); F4/80-BV785 (Biolegend). Leukocytes from the spleen were used for compensation controls (i.e., correction of a signal overlap between emission spectra of different fluorochromes used). Cell viability marker (APC-Cy7, Stemcell Technologies Inc.) was used to exclude dead cells. Forward and side scatter parameters were used to confirm the size and granularity. Analytical cytometry was performed on a 5-laser Becton Dickenson LSR Fortessa II analyzer (BD Biosciences, San Jose, CA) and analyzed using FlowJo software (Tree Star, San Carlos, CA, USA).

### Statistics

Statistical significance was set at *p* < 0.05. All statistical analyses were performed using Prism (version 8) statistical software (Graphpad Software Inc., La Jolla, CA, USA). Results were presented as mean ± standard error of the mean. Box/scatter or violin/scatter configurations were used to show the biological variability when illustrative. Independent sample *t*-test was employed to evaluate the difference between two groups. Tissue and data from oral cancer mouse models were never directly compared; tumor-bearing mice were only compared to time-matched (i.e., equal time post inoculation) sham mice. Two-way and Three-way Analysis of variance (A*NOVA*) repeated measures was employed to evaluate the difference between groups regarding time, sex, and treatment for behavior, tumor size, and weight loss. Two-way A*NOVA* was employed to evaluate the difference between groups regarding time, sex, and/or treatment for proteomic and cytometric evaluation. To adjust for multiple comparisons, the *post-hoc* Holm-Sidak test statistic was employed. Kruskal-Wallis test was used as a non-parametric test for statistical differences in relative gene expression distribution in sensory neurons between treatment groups.

## Results

### Immunogenic properties of cancer cell lines

**Figure 1:**
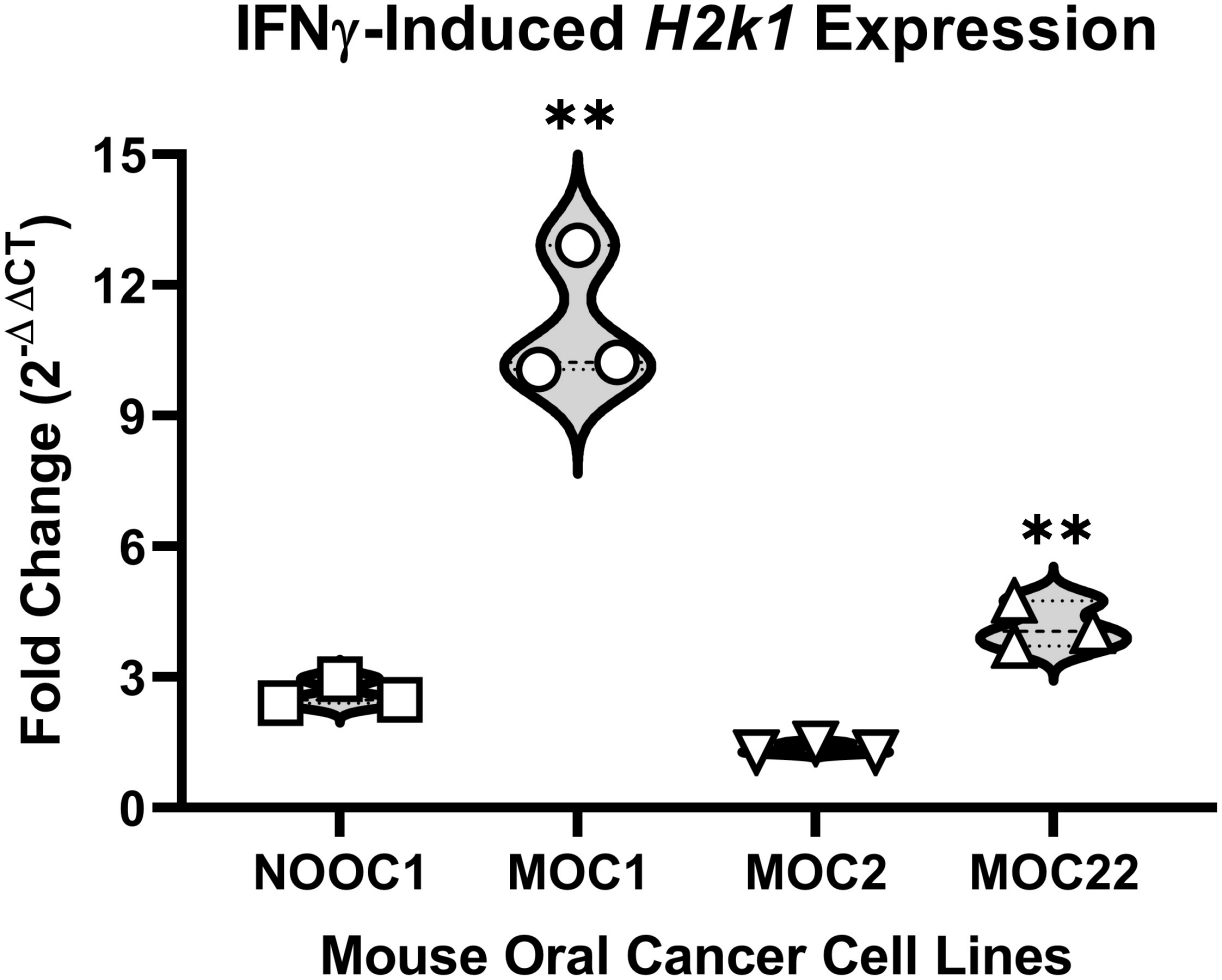
MHC class I expression of mouse oral cancer (MOC) lines. MHC class I transcription factor *H2k1* expression after 24-h stimulation with either vehicle (0.01% BSA) or 100 ng/ml interferon gamma (IFNγ). Cycle threshold values were normalized to housekeeping gene, beta Actin, and reported as fold change (2^−ΔΔCT^) from vehicle treatment. Independent *t-test*, **p < 0.01.

To first determine the immunogenicity of different cell lines, we quantified inducible genes encoding murine major histocompatibility complex (MHC) class I molecules in four mouse-derived cell lines (MOC1, MOC2, MOC22, NOOC1) in response to IFN-γ exposure (24, 25). Three different passages of each line were grown to 50% confluency and then treated with either 100 ng/mL IFN-γ or vehicle (0.1% BSA) for 24 h. Cells were then collected and probed for MHC class-I transcription factor, *H2k1*, using RT-qPCR; relative expression was normalized to housekeeping gene, beta-actin. All samples were run in triplicate and all statistical comparisons were done within cell line to vehicle control. We found that MOC1 cells had an average 12-fold increase in *H2k1* compared to vehicle treated cells [*t*(4) = 15.38, *p* = 0.0001]. MOC22 also significantly responded to IFN-γ stimulation but to a lesser degree with a 4-fold change in *H2k1* expression [*t*(4) = 10.90, *p* = 0.0004]. However, there was no significant change in *H2k1* expression in MOC2 or NOOC1 cells in response to IFN-γ stimulation compared to vehicle stimulation [MOC2: *t*(4) = 2.04, *p* = 0.1104; NOOC1: *t*(4) = 1.70, *p* = 0.1650] (Figure 1). To investigate the impact of immunogenicity on oral cancer pain, we chose to use MOC1 and MOC2 cell lines in an orthotopic transplant model.

### MOC1 and MOC2 tumor progression drives weight loss and orofacial nociceptive behavior in a sex-specific manner

**Figure 2:**
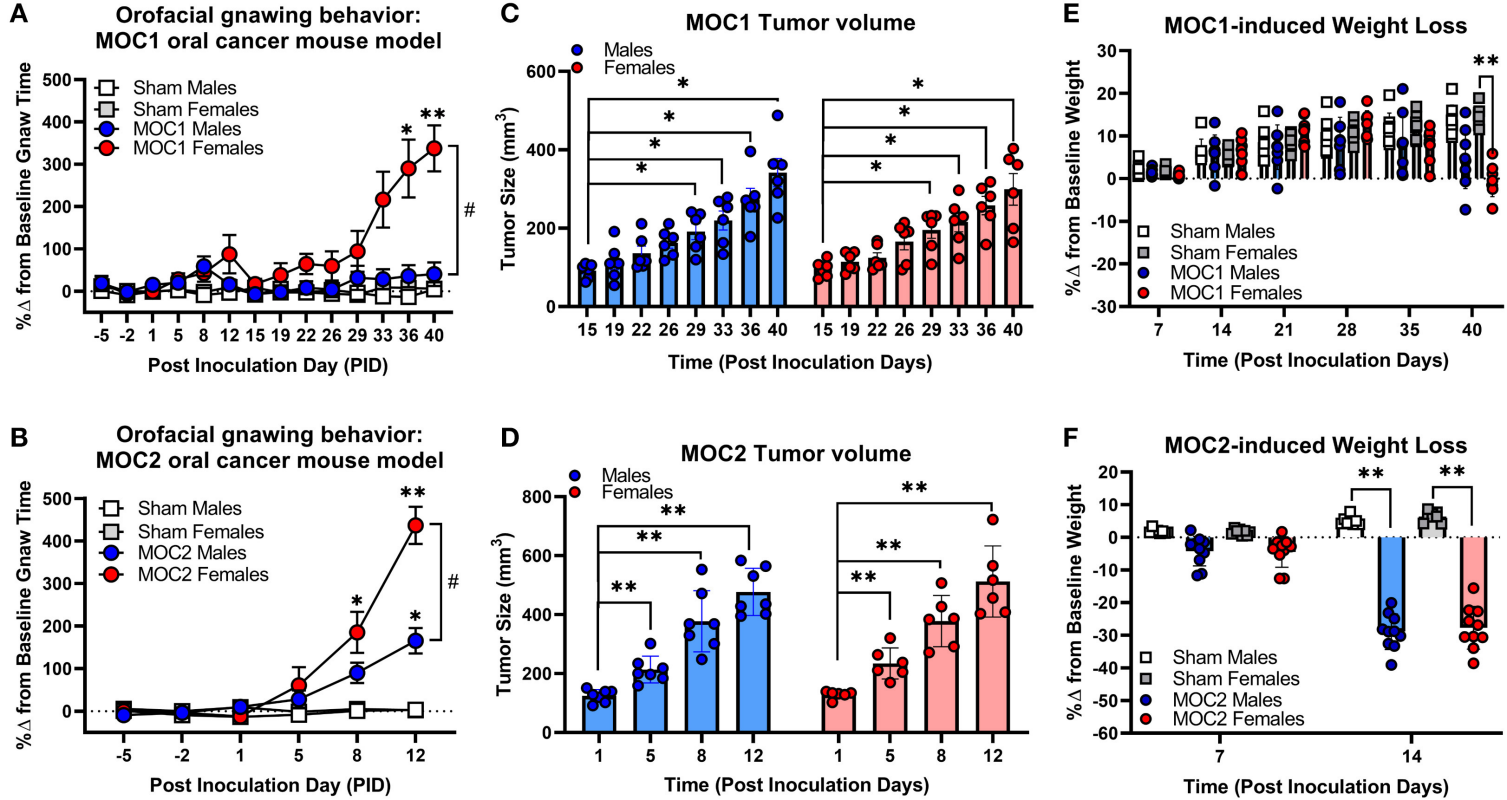
Sex-specific oral cancer-induced nociceptive behavior and weight loss during tumorigenesis. Orofacial nociceptive behavior was measured in male and female sham and MOC1 and MOC2 tumor-bearing mice during tumorigenesis. Two weeks of baseline orofacial nociceptive behavior was acquired prior to inoculation. Mice were inoculated at PID0. Quantitative analysis of the percent change in baseline gnaw-time in sham mice (1:1 matrigel and cell culture media, *n* = 8/sex, squares), or MOC1 **(A)** or MOC2 **(B)** tumor-bearing mice (*n* = 10/sex, circles). Three-Way ANOVA Repeated Measures, time by treatment *p < 0.05, **p < 0.01; sex by time ^#^*p* < 0.05. Orthotopic allograft tumor size was measured for MOC1 **(C)** starting at post-inoculation day (PID) 15 (*n* = 6/sex) and MOC2 **(D)** starting at PID1 (*n* = 6 female, 7 males). Tumor area was measured by caliper and volume calculated as an ellipsoid (V = 4/3π × L × W × H). Two-way ANOVA Repeated Measures, time effect *p < 0.05, **p < 0.01. Allograft-induced weight loss was measured weekly in MOC1 (*n* = 8/sex) **(E)** and MOC2 **(F)** tumor-bearing mice (*n* = 10/sex) and compared to sham-treated mice (*n* = 8/sex). Three-Way ANOVA Repeated Measures, time by treatment *p < 0.05, **p < 0.01.

To initially assess the nociceptive impact of these oral cancer transplant models, we first quantified nociceptive behavior in response to MOC1 and MOC2 tumor progression within the tongue over time as well as the tongue tumor size and cancer-induced weight loss. Nociceptive behavior was measured using the dolognawmeter assay in which gnaw-time is a validated index of orofacial nociception (20) (Figures 2A,B). Tongue tumor volume was measured by calipers under light anesthesia. MOC1 tumors were not visually detectable until PID16, whereas, given the aggressive nature of MOC2, tumor size measurements began on PID1 (Figures 2C,D). Body weight was measured weekly starting at the day of inoculation (Figures 2E,F). Due to differential tongue tumor growth rates between the models, tumor-bearing mice were only statistically compared time-matched sham (i.e., Matrigel only) mice and powered to detect differences between the sexes. For the MOC1 model, a sex difference in orofacial nociceptive behavior was observed during MOC1-induced tumorigenesis [treatment by sex by time, *F*_(11,352)_ = 4.355, *p* < 0.0001]. MOC1-tumor-bearing female mice, but not male mice, exhibited significantly longer gnaw-times compared to sex-matched sham mice at PID36 (*p* = 0.024) and PID40 (*p* = 0.024) (Figure 2A). There was a slow but significant increase in tongue tumor size from starting at PID29 for both sexes (*p* = 0.020) compared to sham; no interaction between time and sex was identified [*F*_(7,70)_ = 0.443, *p* = 0.872] (Figure 2C). Additionally, MOC1 tumor progression resulted in a significant cancer-induced weight loss at PID40 in tumor-bearing female mice compared to PID40 female sham mice [*F*_(5,140)_ = 3.727, *p* = 0.005] (Figure 2E).

The MOC2 model also demonstrated a sex difference in orofacial nociceptive behavior during tumorigenesis [treatment by sex by time, *F*_(5,170)_ = 5.429, *p* = 0.0001] compared to sham mice. Tumor-bearing female mice exhibited significantly longer gnaw-times compared to female sham mice at PID8 (*p* = 0.018) and PID12 (*p* < 0.0001), while tumor-bearing male mice only exhibited significantly longer gnaw-times compared to male sham mice at PID12 (*p* < 0.0001) (Figure 2B). There was also a significant difference in the percent change in gnaw-time between male and female tumor-bearing mice at PID12 (*p* = 0.0016). There was rapid, significant increase in MOC2 tongue tumor size by PID5 (*p* = 0.009) for both sexes; no interaction between time and sex was identified [*F*_(3,33)_ = 0.209, *p* = 0.889] (Figure 2D). Lastly, MOC2 tumor progression resulted in a significant cancer-induced weight loss at PID14 (*p* < 0.0001) compared to sex- and time-matched sham mice for both sexes; no interaction between time and sex was identified [*F*_(1,32)_ = 0.414, *p* = 0.525] (Figure 2F).

### MOC1 and MOC2 tumor progression evokes changes in pain-related genes in the TG and tongue innervating sensory neurons

Next, we sought to assess specific cancer-induced changes in pain-related gene expression during both MOC1 and MOC2 tumor progression. We used quantitative RT-PCR to measure global changes in pain-related gene expression at the whole trigeminal ganglia (TG) level. However, the ganglion is comprised of neurons projecting to all facets of the head as well as multiple cell types beyond neurons (e.g., satellite glial cells, immune cells); in the presence of injury (e.g., tumor growth), ganglionic sympathetic sprouting (26) and immune cells infiltration (27) can occur, both of which might drive intraganglionic communication and changes in transcriptomics that are not indicative of what is happening specifically tumor-associated neurons. Therefore, we also assessed relative gene expression in specifically tongue-innervating primary afferent neurons using retrograde labeling and single cell PCR. We have previously identified a role for the sensory neuropeptide, calcitonin gene-related peptide (CGRP), and chemosensitivity receptor, transient receptor vanilloid channel 1 (TRPV1), in oral cancer pain (19, 22). Additionally, given the differences in immunogenicity and tumor growth *in vivo*, we sought to understand the potential contribution of nerve injury and subsequent neuropathic pain (9) for each model. Therefore, for this concise characterization, we chose to focus on cancer-induced changes in the pain-related genes *Calca* and *Trpv1*, as well as the injury marker, *Atf3*. Intact TG from MOC1- (PID40) and MOC2- (PID14) bearing mice were probed for the target genes using qPCR; relative fold change in expression was calculated to respective time and sex-matched sham mice for both groups.

**Figure 3:**
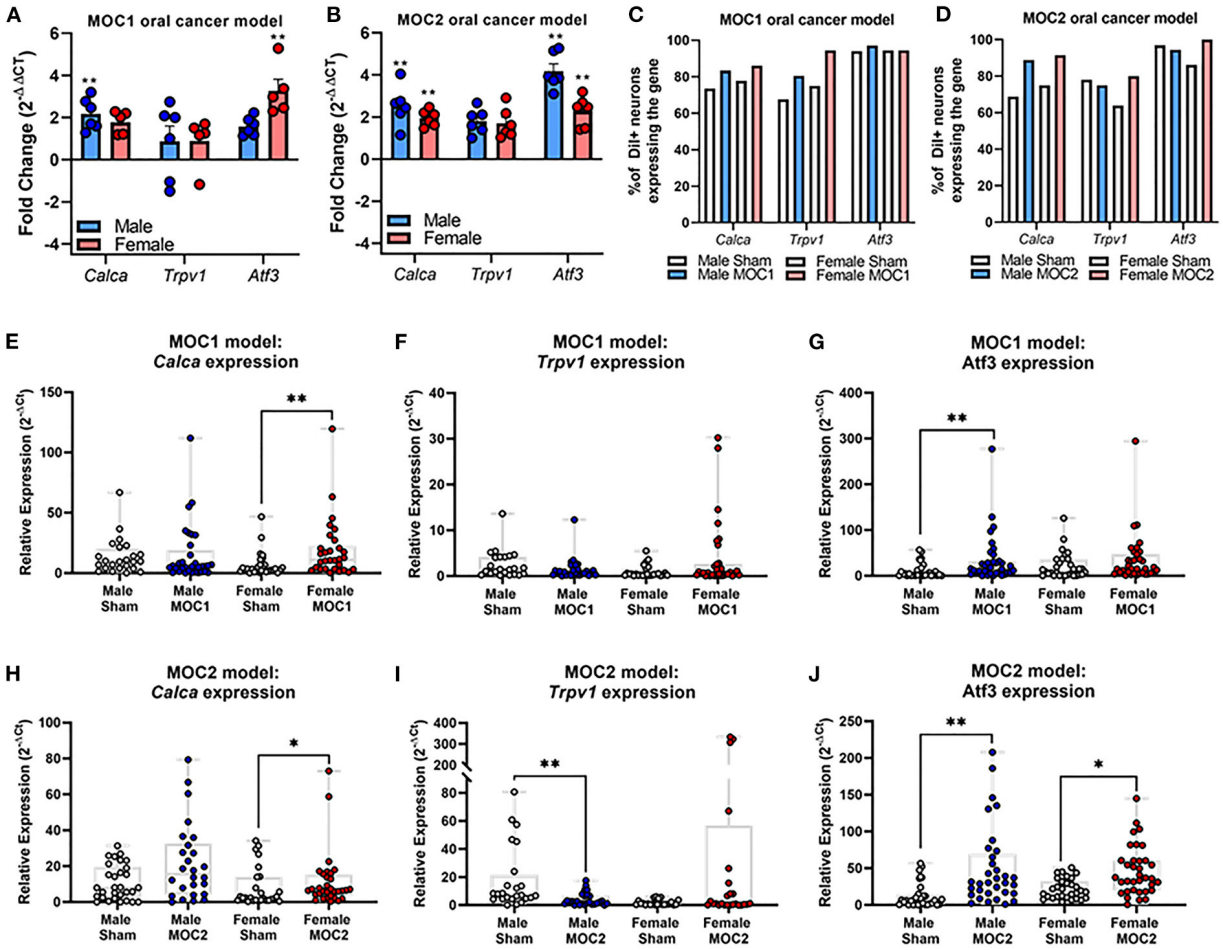
Oral cancer-induced changes in pain-related gene expression. Quantitative PCR was performed to assess changes in *Trpv1, Calca*, and *Atf3* in whole ganglia from **(A)** sham and MOC1 tumor-bearing mice at PID40 as well as **(B)** sham and MOC2 tumor-bearing mice at PID14. *Gapdh* was used as an internal control. Significance was determined by comparing delta CT values between treatment groups within sexes (*n* = 5–6/sex/group, Independent t-test, **p < 0.01). To assess changes in target genes at the single cell level, retrogradely labeled tongue-innervating TG neurons were manually picked to perform single-cell PCR (*n* = 36 neurons from 3 mice/sex/treatment). Gene expression was calculated for relative mRNA expression in each cell for each gene. *GusB* was used as an internal control. The percentage of neurons expressing the target genes was calculated for **(C)** sham and MOC1 tumor-bearing mice at PID40 as well as **(D)** sham and MOC2 tumor-bearing mice at PID14. **(E–J)** Relative mRNA expression values from single-cell PCR for each gene are presented as max to min with all points shown. Expression intensities for each gene between groups was compared using non-parametric Kruskal-Wallis with Bonferroni *post hoc* test. *p < 0.05, **p < 0.01.

In the MOC1 model at the whole ganglia level (Figure 3A), there was no significant interaction between sex and treatment for *Calca* [*F*_(1,18)_ = 0.251, *p* = 0.622] or *Trpv1* [*F*_(1,18)_ = 0.087, *p* = 0.771]. However, when assessing the sexes individually, there was a 2-fold increase in *Calca* expression in MOC1 tumor-bearing male mice compared to male PID40 sham (*p* = 0.014). For *Atf3* expression, there was a significant interaction between sex and treatment [*F*_(1,18)_ = 8.034, *p* = 0.011]; tumor-bearing female mice had a 3.5-fold increase in *Atf3* expression compared to female PID40 sham mice (*p* < 0.0001). In the MOC2 model (Figure 3B), there was no significant interaction between sex and treatment for *Calca* [*F*_(1,19)_ = 0.520, *p* = 0.480] or *Trpv1* [*F*_(1,19)_ = 4.326, *p* = 0.051], similar to the MOC1 model. However, when assessing the sexes individually, there was 2-fold increase in *Calca* expression in tumor-bearing male (*p* = 0.005) and female (*p* = 0.037) mice compared to sex-matched sham PID14 mice. There was no effect of treatment within sexes for *Trpv1* expression in whole TG from either male (*p* = 0.063) or female (*p* = 0.366) mice. For *Atf3* expression, there was a significant interaction between sex and treatment [F_(1,19)_ = 4.912, *p* = 0.039]. There was a 4- and 2.5-fold increase in *Atf3* expression in tumor-bearing male (*p* < 0.0001) and female (*p* = 0.002) mice, respectively, compared to sex-matched PID14 sham mice. While there was no difference in *Atf3* expression between tumor-bearing male and tumor-bearing female mice (*p* = 0.728), there was significantly lower *Atf3* expression in male PID14 sham mice compared to female PID14 sham mice (*p* = 0.002).

To assess tongue-innervating neuron-specific expression, ganglia from retrogradely labeled mice were dissociated, and individual neurons were collected for single-cell analysis based on fluorescence. RNA from individual DiI-labeled neurons (*n* = 36 neurons from 3 mice *per sex* per treatment group) were evaluated as percent of neurons expressing either *Calca, Trpv1* and *Atf3* (Figures 3C,D) as well as relative expression (Figures 3E–J) using single cell qPCR analysis.

In the MOC1 model (Figure 3C), *Calca* was expressed in about 75% of neurons from sham PID40 mice (26/36 male, 28/36 female) and 85% of neurons from MOC1-tumor bearing mice (30/36 male, 31/36 female), however the relative expression was significantly greater in only tumor-bearing female mice compared to sham (*p* = 0.013, Figure 3E). *Trpv1* was expressed in about 70% of neurons from sham mice (24/36 male, 27/36 female) and >80% of neurons from MOC1-tumor bearing mice (29/36 male, 34/36 female, Figure 3C). There was no significant difference in relative expression from tumor-bearing male mice (*p* = 0.080) or tumor-bearing female mice (*p* = 0.154) compared to sex-matched PID40 sham (Figure 3F). While *Atf3* was expressed in the majority of neurons from sham mice (34/36 male, 34/36 female) and MOC1-tumor bearing mice (35/36 male, 34/36 female, Figure 3C), the relative expression was significantly greater in neurons from male (*p* = 0.008) but not female (*p* = 0.467) tumor-bearing mice compared to neurons from sex-matched sham (Figure 3G).

In the MOC2 model, *Calca* was expressed in about 70% of neurons from sham mice (23/36 male, 27/36 female) and 90% of neurons from MOC2-tumor bearing mice (32/36 male, 33/36 female, Figure 3D), and similar to the MOC1 model, the relative expression was significantly greater in tumor-bearing female mice compared to female PID14 sham (*p* = 0.048, Figure 3H). *Trpv1* was expressed in more than 75% of neurons from sham mice (28/36 male, 31/36 female) and MOC2-tumor bearing mice (26/36 male, 23/36 female, Figure 3D). However, contrary to the MOC1 model, the relative expression was significantly less in neurons from tumor-bearing male mice compared to sham (*p* = 0.001, Figure 3I). Lastly, while *Atf3* was expressed in the majority of sham (33/36 male, 36/36 female) and MOC2 tumor-bearing (33/36 male, 29/36 female, Figure 3D) mice, the relative expression was significantly greater in neurons from male tumor-bearing mice (*p* = 0.001) and female tumor-bearing mice (*p* = 0.034) compared to neurons from sex-matched PID14 sham (Figure 3J).

### MOC1 and MOC2 tumor progression drives changes in pain-related protein expression in tongue innervating sensory neurons

**Figure 4:**
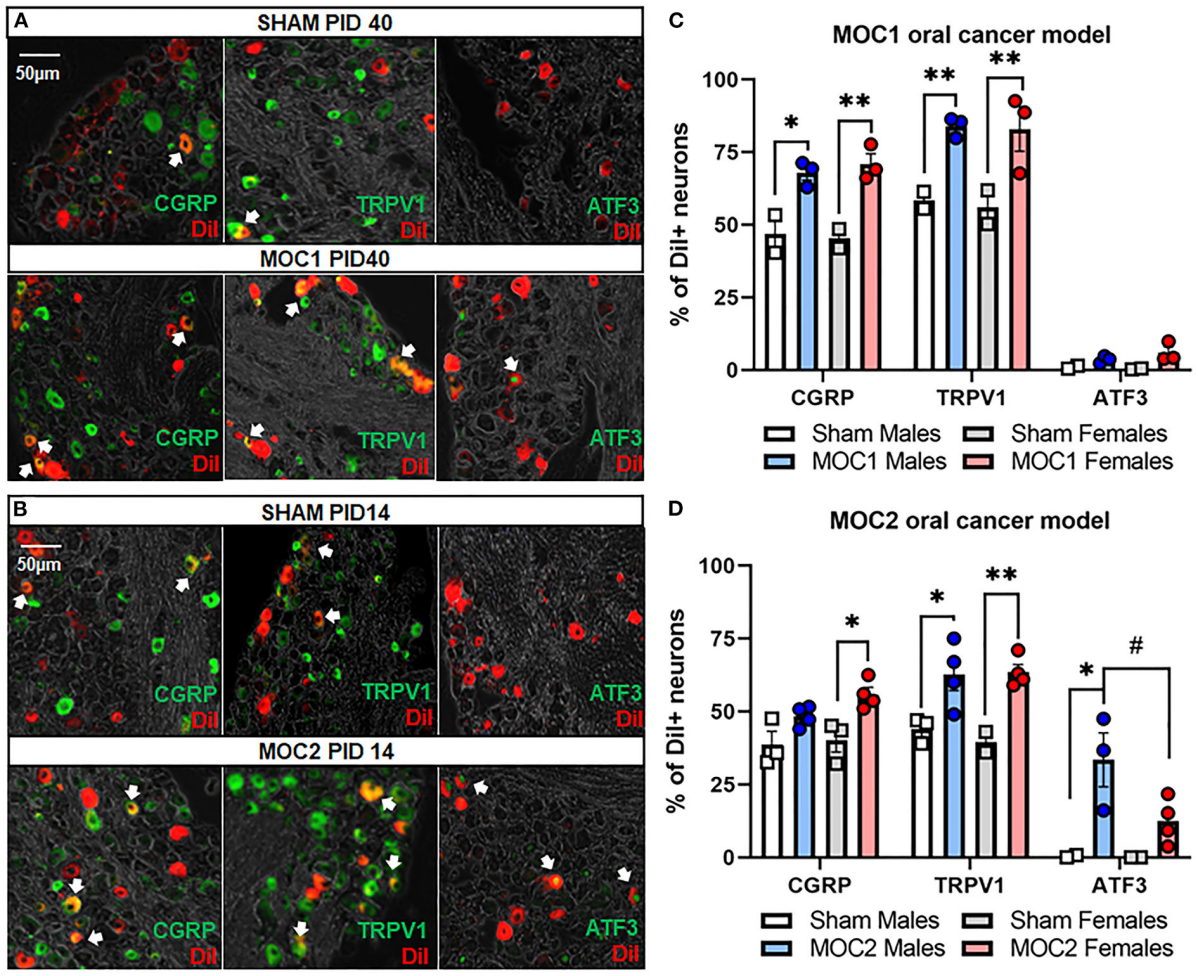
Oral cancer-induced changes in pain-related protein expression. Each TG tissue sections from each retrogradely labeled mouse was stained with antibodies specific for either TRPV1, CGRP or ATF3. Representative immunostaining of all three markers in retrogradely labeled TG tissue from **(A)** sham and MOC1 mice at PID40 as well as **(B)** sham and MOC2 mice at PID14. **(C,D)** The percentage of DiI-labeled neurons expressing each marker was quantified from 2–4 animals per treatment group. Data are presented as mean +/- SEM of percentage of neurons expressing each marker. Two-way ANOVA, Treatment effect: ^*^p < 0.05, ^**^p < 0.01, sex effect: #*p* < 0.05.

Next, we sought to assess cancer-induced changes in pain-related protein expression in tongue-innervating primary afferent neurons during MOC1 and MOC2 tumor progression. TG from retrogradely labeled MOC1- (PID40) and MOC2- (PID14) bearing mice and respective sex- and time-matched sham mice were harvested, sectioned, and stained using immunohistochemistry (Figures 4A,B). The percentage of DiI-positive neurons with TRPV1-, CGRP-, or ATF3- immunoreactivity (IR) were quantified.

In the MOC1 model (Figure 4C), we found no significant interaction between sex and treatment in CGRP expression [*F*_(1,6)_ = 0.375, *p* = 0.563], TRPV1 expression [*F*_(1,6)_ = 0.018, *p* = 0.899], or ATF3 expression [*F*_(1,6)_ = 1.180, *p* = 0.319]. When assessing the sexes individually, there was a significant increase in the percentage of DiI-positive CGRP-IR neurons and TRPV1-IR in TG sections from MOC1 tumor-bearing male (*p* = 0.009, *p* = 0.002 respectively) and female (*p* = 0.002, *p* = 0.001 respectively) mice and compared to sex-matched PID40 sham mice. However, regarding ATF3-IR, there was no change in expression in MOC1 tumor-bearing male (*p* = 0.773) or female mice (*p* = 0.152) compared to PID40 sham.

In the MOC2 model (Figure 4D), we found no significant interaction between sex and treatment in CGRP expression [*F*_(1,11)_ = 0.587, *p* = 0.460] or TRPV1 expression [*F*_(1,9)_ = 0.365, *p* = 0.561]. When assessing the sexes individually, there was a significant increase in the percentage of DiI-positive CGRP-IR neurons (*p* = 0.032) and TRPV1-IR (*p* = 0.007) in TG sections from MOC2 tumor-bearing female mice compared to female PID14 sham mice. MOC2 tumor bearing male mice had a higher percentage of DiI-positive TRPV1-IR neurons (*p* = 0.047), but not CGRP-IR (*p* = 0.272) neurons, compared to male PID14 sham mice. For ATF3-IR, there was a significant interaction between sex and treatment [*F*_(1,11)_ = 6.195, *p* = 0.030]. TG sections from tumor-bearing male mice had more DiI-positive ATF3-IR neurons compared to tissue from male PID14 sham (*p* = 0.001) as well as compared to tissue from MOC2 tumor-bearing female mice (*p* = 0.032). There was no significant difference in the percentage of DiI-positive ATF3-IR neurons in TG sections from MOC2 tumor-bearing female mice compared to female PID14 sham mice (*p* = 0.250).

### MOC1 and MOC2 tumor progression evoke differential immune infiltrate over time

**Figure 5:**
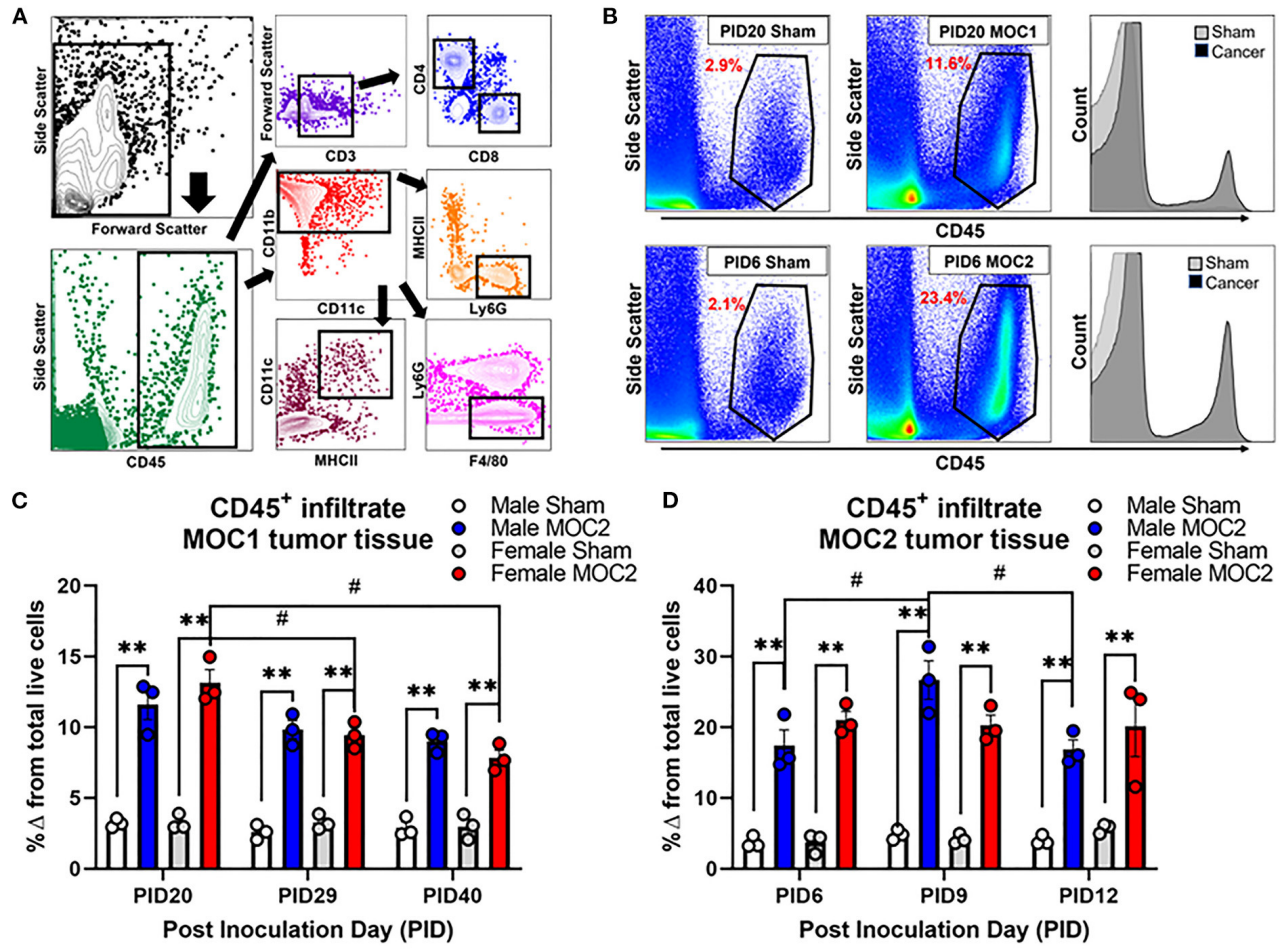
Tumor-associated immune infiltrate during tumor progression. Immune infiltrate in tongue tissue was measured in male and female sham, MOC1 and MOC2 tumor-bearing mice during tumorigenesis. **(A)** Analytical cytometric gating strategy used to characterize immune subpopulations. Following viability gating to isolate live cells (not shown), CD45+ cells were gated into either CD3+/- lymphocytes or CD11b+ myeloid cells. CD3+ cells were further gated into CD4 T helper or CD8 cytotoxic T cells. CD3- cells were further gated into CD19+ B cells or NK1.1+ NK cells. The CD11b+ leukocytes were gated into MHCII+CD11c+ dendritic cells, Ly6G-F4/80+ macrophages, or CD11b+Ly6g+ neutrophils. **(B)** Representative scatter plots showing CD45+ immune cells in tongue tissue from sham and MOC1 tumors at PID20 as well as in sham and MOC2 tumors at PID6. Histograms demonstrate the increase in CD45+ counts in tumor tongue tissue compared to sham. CD45+ immune infiltrate was quantified by analytical cytometry at three different time points in the MOC1 **(C)** and MOC2 **(D)** models. *N* = 3 mice/sex/timepoint. Within sex comparisons were done to assess the effect of treatment (**p < 0.01) and time (#*p* < 0.05) by Two-way ANOVA.

Flow cytometry was performed by the gating strategy defined in Figure 5A to assess immune cell infiltrate into the tongue during MOC1 and MOC2 tumor progression. While there was no significant interaction between time, sex, and treatment in the MOC1 model with regard to CD45^+^ cells (all leukocytes) in the tongue tissue, we did find a significant effect of treatment [i.e., sham vs. MOC1, *F*_(1,12)_ = 424.2, *p* < 0.0001] where there was significantly more CD45^+^ cell infiltrate in MOC1 tumor bearing mice compared to sham at each timepoint (Figures 5B,C). Additionally, when assessing the sexes separately, there was an interaction between treatment and time in female mice only [*F*_(2,12)_ = 9.750, *p* = 0.003]; there was significantly more CD45^+^ immune cells in tongue tissue from female MOC1-tumor bearing mice at PID20 compared to both PID29 (*p* = 0.040) and PID40 (*p* = 0.025) (Figure 5C).

In the MOC2 model, there was no significant interaction between time, sex, and treatment for CD45^+^ cell infiltrate in the tongue tissue, but rather a significant effect of treatment [i.e., sham vs. MOC2 tumor; *F*_(1,12)_ = 201.9, *p* < 0.0001] where there was significantly more CD45^+^ cell infiltrate in MOC2 tumor bearing mice compared to sham (Figures 5B,D). Additionally, when assessing the sexes separately, there was an interaction between treatment and time in male mice only [*F*_(2,12)_ = 4.421, *p* = 0.0364]; there were significantly more CD45^+^ immune cells in the tongue tissue from male MOC2-tumor bearing mice at PID9 compared to both PID6 (*p* = 0.040) and PID12 (*p* = 0.025).

**Figure 6:**
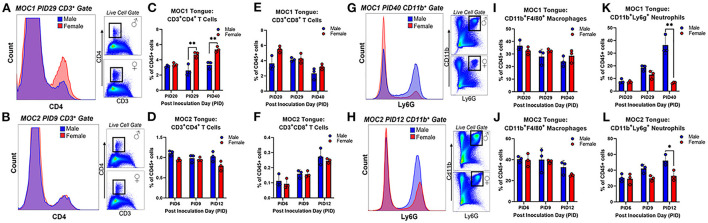
Sex-specific changes in tongue tumor immune microenvironment. Representative histogram and scatter plots showing CD3^+^CD4^+^ T cell populations in **(A)** tongue tissue from sham and MOC1 tumors at post inoculation day (PID) 29 and **(B)** tongue tissue from sham and MOC2 tumors at PID9. The percentage of CD45^+^ cells that were **(C,D)** CD3^+^CD4^+^ T cells and **(E,F)** CD3^+^CD8^+^ T cells were quantified in male and female tumor-bearing mice and compared across 3 timepoints (MOC1: PID20, 29 and 40; MOC2: PID6, 9, 12; *n* = 3/sex/timepoint). Representative histogram and scatter plots showing CD11b^+^Ly6G^+^ neutrophil populations in **(G)** tongue tissue from sham and MOC1 tumors at PID40 and **(H)** tongue tissue from sham and MOC2 tumors at PID12. The percentage of CD45^+^ cells that were **(I,J)** CD11b^+^Ly6G^+^ neutrophils and **(K,L)** CD11b^+^Ly6G^−^F4/80^+^ macrophages were quantified in male and female tumor-bearing mice and compared across 3 timepoints (*n* = 3/sex/timepoint). Two-way ANOVA (sex by time), *p < 0.05, **p < 0.01.

Next, we sought to investigate the immune cell subtypes that comprise the tongue tumor infiltrate between the sexes to begin to elucidate the influence of inflammation on cancer-induced nociceptive behavior. In total we assessed oral cancer-induced changes in putatively defined, macrophages, dendritic cells, neutrophils, T helper cells, cytotoxic T cells, B cells, and natural killer cells. The percentages of total live cells for each population at each timepoint are available for both sham and tumor-bearing mice in tongue tissue (Supplementary Table 1) and draining lymph node tissue (Supplementary Table 2). For this report, we focused on T cells (Figures 6A,B) given their role in tumor immunosurveillance as well as literature support for sex-dependent neuroimmune pain signaling pathways (28, 29). In the MOC1 model, we found a significant interaction between sex and time [*F*_(2,8)_ = 8.289; *p* = 0.011] in the percentage of CD45^+^CD3^+^CD4^+^ helper T cells in the MOC1 tumor tissue; tumor tissue from MOC1-bearing female mice had significantly more CD4^+^ cells at PID29 (*p* = 0.0002) and PID40 (*p* = 0.0003) compared to tumor-bearing male mice (Figures 6A,C). There was a similar percentage of CD45^+^CD3^+^CD8^+^ cytotoxic T cells in tumor bearing male and female mice over time [*F*_(2,8)_ = 0.151; *p* = 0.862] (Figure 6E).

In the MOC2 model, we found no significant differences between the sexes over time for either CD4^+^ cells [*F*_(2,8)_ = 2.169; *p* = 0.177] or CD8^+^ cells [*F*_(2,8)_ = 0.151; *p* = 0.862] (Figures 6B,D,F). In addition to T cells, we investigated infiltration of putative CD11b^+^F4/80^+^ macrophages and CD11b^+^Ly6G^+^ neutrophils in both models based on previous cancer pain literature indicating a role for these cell types in sex-dependent nociception (28) and anti-nociception (23, 30), respectively. While there were no differences detected between sexes over time with regard to putative macrophages in either MOC1 [*F*_(2,8)_ = 3.764; *p* = 0.070] or MOC2 [*F*_(2,8)_ = 0.411; *p* = 0.677] models (Figures 6I,J), we did detect a significant interaction between sex and time in putative neutrophil infiltration (Figures 6G,H,K,L). MOC1 and MOC2 tumor-bearing male mice had a significantly higher percentage of CD45^+^CD11b^+^Ly6g^+^ cells compared to female tumor-bearing mice at PID40 [*F*_(2,8)_ = 30.33, *p* = 0.0002] and PID12 [*F*_(2,8)_ = 4.636, *p* = 0.046] respectively.

## Discussion

The results presented here indicate that the immunogenicity of the cell line chosen to generate an orthotopic oral cancer transplant model can have a distinctive impact on the target tissue microenvironment, particularly affecting the immune and sensory nervous systems. We profiled the immunogenicity of four different carcinogen-induced mouse oral cancer cell lines by measuring the inducible levels of MHC class I molecule gene expression in response to IFN-γ. Production of INF-γ is regulated by the immune response to tumor-secreted cytokines (31). Downregulation of cell surface MHC class I molecules, which are necessary for CD8^+^ T-cell detection of tumor cells, is associated with tumor progression and poor survival in patients with HNSCC (32, 33). Therefore, Therefore, the differing MHC class I regulatory responses of these cell lines to INF-γ could contribute to their aggression (24). We found that MOC1 had the largest increase in class I transcription factor *H2k1* in response to IFN-γ, whereas MOC2 showed no significant response. Furthermore, we showed that MOC1 cells had slower growth vs. MOC2 when inoculated orthotopically in immunocompetent mice; quantification of tumor size over time indicated that in the MOC1 model, measurable tumor growth was not detectable until 4 weeks after inoculation whereas in the MOC2 model, there was a significant increase in tumor size by day 5. These results are consistent with a previous study by Judd and colleagues which demonstrated that MOC1 has elevated levels of inducible MHC class I proteins compared to MOC2 cells and that MOC1 grew more slowly subcutaneously in immunocompetent mice (34). IFN-γ signaling in cancer cells also includes regulation beyond the induction of MHC class I molecules. The IFN-γ induced inflammatory cascade can summon immune-related cell types (35) as well as stimulate upregulation of tumor growth molecules [i.e, PD-*L1* checkpoint molecule (36)] algogenic proteins that may impact tumor cell-neuron communication and evoke nociception (35), such as cathepsin (37), VEGF (38), and nitric oxide synthase (39). Additional investigation is needed to understand how immunogenicity and cancer-secreted molecules may impact growth rate and nociceptive behaviors observed. Currently, our findings suggest that components of adaptive immunity are able to suppress tumor growth *in vivo*, a response which may be a critical regulator in understanding the development of oral cancer pain.

To investigate the relationship between immunogenicity, tumor progression and pain, we utilized the dolognawmeter orofacial pain device and assay to quantify the nociceptive behavior in response to MOC1 and MOC2 growth. We and others have previously utilized the dolognawmeter assay to measure oral cancer-induced nociceptive behavior in both acute (40, 41) and chronic (13, 23, 40) oral cancer pain models, however, the dolognawmeter assay and device has not been used previously in a syngeneic transplant oral cancer model. In this study, we found the onset and progression of tumor-induced nociceptive behavior consistent with the tumor size development and cancer-induced weight loss in both models. However, given the low immunogenic quality of MOC2, the tumor growth and corresponding evoked nociceptive behavior and weight loss had a rapid onset, with tumor growth detectable at PID1 and significant changes in nociceptive behavior at PID8. Sex differences in oral cancer-evoked nociceptive behavior using mouse models (22, 23, 30) have also been previously reported but are still debated in the clinical literature (12, 23, 42, 43). In the MOC1 model, despite similar tumor size over time, only tumor-bearing female mice demonstrated significant tumor-evoked nociceptive behavior over time compared to sex-matched shams. Consistently, there was only significant cancer-induced weight loss in female mice at PID40 compared to sex-matched sham mice. In the MOC2 model, tumor-bearing female and male mice both demonstrate a significant increase in gnaw-time compared to their respective sex-matched sham groups; however, females demonstrated significantly more evoked nociceptive behavior than males. There was no difference detected in tumor size or cancer-induced weight loss over time between the sexes. We previously hypothesized that endogenous opioid anti-nociception mechanism contributed to the decreased nociceptive behavior in tumor-bearing male mice (23); however, this hypothesis has not yet been explored in the MOC models. While our results are consistent with previous findings using the dolognawmeter assay, our report is limited by using only one behavioral assay. Additional nociceptive behaviors have been validated previously on animal models of oral cancer pain such as conditioned place preference (22, 30, 44) (CPP), facial von Frey (45), and meal analysis (44, 46); however, sex differences in orofacial nociception have only been previously documented using the CPP (22, 30).

Oral cancer-induced changes in pain-associated mediators have been previously studied in acute, xenograft transplant and carcinogen-induced oral cancer pain mouse models (16, 40, 45, 46). For investigation into the syngeneic allograft transplant model, we chose to focus on three nociception-related proteins expressed on primary afferent sensory neurons, TRPV1, CGRP, and ATF3 and their associated genes, *Trpv1, Calca, Atf3*. Cancer-induced increases in TRPV1 and CGRP protein expression in the TG neurons have been previously reported in association of nociception in both rat (47) and mouse (19, 22) oral cancer models. We found, within the MOC1-and MOC2- models, that *Calca* gene expression and CGRP protein in tongue-innervating neurons are increased in primarily female mice, an effect which occurs along with the sex differences in nociceptive behavior. While ATF3 protein expression was previously investigated in a orthotopic xenograft mouse model and found to not be present in TG from tumor-bearing male mice (44), other cancer pain models [e.g., bone cancer (48), pancreatic (49)] have shown increased expression of ATF3 in primary afferent neurons innervating the tumor microenvironment. We found cancer-induced increase in relative *Atf3* gene expression in MOC1 males and MOC2 males and females compared to sex-matched sham treated mice as well as a cancer-induced increase in ATF3-IR in MOC2-tumor bearing males only compared to male PID14 sham mice. At the whole ganglia level, there was significantly less *Atf3* gene expression in male PID14 sham mice compared to female PID14 sham mice. While the underlying cause is unknown, this may be due to sex-specific prolonged injury response to sham injection in female mice. At the single cell level, we also unexpectedly found that the majority of tongue-innervating TG neurons from both sham and tumor-bearing mice expressed *Atf3*, likely from the trigeminal ganglia dissociation process; however, the relative expression was significantly higher in MOC1 tumor-bearing males and in MOC2 tumor- bearing male and female mice compared to sham suggesting that cancer does induce an injury response greater than that of experimental processing. We also found significantly increased ATF3-IR in retrograde labeled TG neurons from MOC2 tumor-bearing male mice compared to male PID14 sham but no difference in ATF3-IR in MOC1-tumor bearing mice compared to PID40 sham mice. Rapid onset of tumor burden in the MOC2 model may explain the tumor-induced injury response in this model; by comparison, the slow onset of growth in the MOC1 model may allow the sensory nervous system to adapt to the tumor microenvironment, avoiding sustained injury. However, this hypothesis does not explain the sex-specific increase in ATF3-IR in the MOC2 model. While previous studies have reported male-only upregulation of *Atf3* in dorsal root ganglia following nerve injury (50, 51), the underlying mechanisms and overall effect remains to be studied. Though we did not detect a significant change in *Trpv1* expression at the whole ganglia level, we did find a significant reduction in *Trpv1* relative expression in tongue-innervating TG neurons from MOC2 tumor-bearing male mice compared to male PID14 shams. A reduction in pain-related gene expression in response to trigeminal nerve injury has been previously reported (52), suggesting that tumor-induced nerve injury may be the driving force for this loss of *Trpv1* expression in tumor-bearing male mice. However, since levels of transcription do not inherently correlate with protein expression, we also investigated the protein expression and found that TRPV1-IR was significantly increased in retrograde-labeled TG neurons in both sexes in both the MOC1 and MOC2 models. Together, these results suggest that both tongue tumor models induce plasticity in nociception-related genes and proteins in tongue-innervating neurons.

Characterization of the tumor immune microenvironment in syngeneic oral cancer mouse models, including MOC1 and MOC2 cell lines, has been done previously (34, 53). Our experimental design differs from previous reports by using orthotopic inoculation and by following a cytometric time course as it related to nociceptive behavior. One major limitation of this experiment is the limit sample size of 3 per sex per timepoint; the experiment was not fully powered to detect differences between sexes in all immune cell subtypes reported, therefore we only focused on putative T cells, macrophages, and neutrophils. Immune infiltrate in MOC1 and MOC2 tumor tissue was evaluated starting at PID20 and PID6, respectively. Despite the lack of cancer-induced nociception and cancer-induced weight loss at these timepoints, there was significant CD45^±^ immune infiltrate in both MOC1 and MOC2 tumor tissue suggesting that inflammatory cells in the tumor microenvironment may not influence nociception and weight loss as much as tumor burden. Given the immunogenic nature of MOC1 and consistent with previous literature (34), there were substantially more CD4^+^ and CD8^+^ T lymphocytes present in MOC1 tumor tissue when qualitatively compared to MOC2 tumor tissue. There were significantly more CD4^+^ T cells in MOC1 tumor tissue from female mice compared to male mice at PID29 and PID40. Previous literature using other non-cancer pain models [e.g., migraine (54), chemotherapy-induced neuropathy (29), nerve injury (28)] have demonstrated a sex-specific mechanism for T cell infiltrate in primary afferent hypersensitivity, however future studies are needed to tease apart the role of T cell signaling in oral cancer pain. While we did not detect any differences in CD11b^±^F4/80^±^ macrophage presence in tumor tissue over time or between models, we did find significantly more CD11b^±^Ly6g^±^ neutrophils in MOC1 tumor tissue from male mice at PID40 and MOC2 tumor tissue from male mice at PID12 compared to the sex- and time-matched shams. Increased neutrophil presence in the tumor tissue correlates with the lack of cancer-induced weight loss and nociception in MOC1-tumor bearing male mice. This data is consistent with our previous reports of an increased neutrophil infiltrate in 4NQO-induced tumors in male mice (23) which was shown to contribute to an endogenous opioid-mediated anti-nociceptive mechanism (23, 30). Previous studies have also demonstrated that TRPV1 activation can modulate μ-opioid receptor phosphorylation and delay desensitization (55, 56) suggesting TRPV1 upregulation in these models may augment endogenous opioid signaling. However, orofacial nociception and weight loss was still evident in MOC2-tumor bearing male mice, likely due to larger tumor burden and nerve injury present in this model. Previous research has shown significant levels of CD11b^+^Gr1^+^ cells in syngeneic tumors, but no time- or sex-dependent differences Gr1^+^ infiltrate in MOC1 and MOC2 tumors were noted (34). The differences found in our study may be due in part to the limited sample size as well as the inoculation site, since the immune microenvironment has been shown to be markedly different between orthotopic and subcutaneous oral squamous cell tumors (53). Lastly, given that macrophages represented a large majority of myeloid derived cells in the tumor microenvironment in response to both immunogenic and non-immunogenic tumor growth, additional characterization of the M1 and M2 macrophage phenotype during nociceptive onset and progression could be critical in understanding of tumor neuroimmune interactions.

There are several oral cancer pain hypotheses that this study did not address. One theory incorporates mediators or exosomes released by cancer cells that act on tumor-innervating nociceptors to drive sensitization (5, 57–59). While we investigated the nociceptive neuron plasticity through the expression of TRPV1 and CGRP expression, we did not evaluate mediators released specifically from cancer cells that could affect nociceptive signaling. Other cancer pain-related mediators have been previously well-described [e.g., BDNF (44, 46), NGF (13, 60), PAR2 (22, 40)]. We acknowledge that the data presented in this manuscript is hypothesis-driving and a mechanism linking cancer-induced nociception, inflammatory infiltrate, and cancer-induced sensory neuron plasticity has yet to be determined. This study found that these cell lines with their differing immunogenicity evoke unique neuroimmune interactions, tumor progression timelines, and pain development profiles. Therefore, any experimental design including these lines must consider cell line immunogenicity, *in vivo* growth rate, and engagement with the microenvironment, along with inoculation location, to optimize the translatability of the results and incorporate the distinct features of each model.

## Data availability statement

The original contributions presented in the study are included in the article/supplementary material, further inquiries can be directed to the corresponding author/s.

## Ethics statement

The animal study was reviewed and approved by University of Pittsburgh Institutional Animal Care and Use Committee.

## Author contributions

NH conducted experiments, completed blinded data analysis, and wrote the manuscript. LM and MA designed research, conducted experiments, and edited the manuscript. MY conducted experiments and completed blinded data analysis. The allocation keys were held by NS who also designed the research, conducted experiments and assisted in the writing and editing of the manuscript. All authors listed contributed substantially to the work.

## Funding

This work was supported by a grant from the National Institutes of Health (R00DE028019, NS).

## Conflict of interest

JD fabricates dolognawmeter™ assay devices through Gnatheon Scientific LLC. The remaining authors declare that the research was conducted in the absence of any commercial or financial relationships that could be construed as a potential conflict of interest.

## Publisher's note

All claims expressed in this article are solely those of the authors and do not necessarily represent those of their affiliated organizations, or those of the publisher, the editors and the reviewers. Any product that may be evaluated in this article, or claim that may be made by its manufacturer, is not guaranteed or endorsed by the publisher.
