# Supplementary material for: The impact of tumor immunogenicity on cancer pain phenotype using syngeneic oral cancer mouse models
Source: Front Pain Res (Lausanne). 2022 Sep 12;3:991725. doi: 10.3389/fpain.2022.991725 (PMC9512086; doi:10.3389/fpain.2022.991725)
Supplement: Supplementary file 1 [file Table_1.docx]

**Supplemental Table 1: Immune cell subtypes into tongue tissue**

| **Sample** | ***CD45^+^** | **Myeloid Derived** | | | **Lymphocyte** | | | |
| --- | --- | --- | --- | --- | --- | --- | --- | --- |
|  |  | **Cd11c^+^** | **Ly6g^+^** | **F4/80^+^** | **CD4^+^** | **CD8^+^** | **CD19^+^** | **NK1.1^+^** |
| **Sham Male** | | | | | | | | |
| PID 20 | 3.2 ± 0.3 | 6.9 ± 0.3 | 1.0 ± 0.2 | 38.3 ± 6.4 | 0.8 ± 0.4 | 1.2 ± 0.3 | 0.6 ± 0.3 | 0.5 ± 0.1 |
| PID 29 | 2.6 ± 0.5 | 7.5 ± 0.6 | 1.0 ± 0.2 | 29.3 ± 4.0 | 0.6 ± 0.2 | 5.3 ± 1.5 | 0.8 ±0.3 | 0.5 ± 0.05 |
| PID 40 | 2.9 ± 0.7 | 3.1 ± 2.4 | 0.8 ± 0.6 | 27.1 ± 9.2 | 0.5 ± 0.3 | 4.1 ± 1.8 | 0.8 ± 0.3 | 0.3 ± 0.4 |
| **Sham Female** | | | | | | | | |
| PID 20 | 3.3 ± 0.5 | 10.1 ± 1.6 | 0.98 ± 0.2 | 37.8 ± 4.6 | 1.2 ± 0.7 | 1.9 ± 0.6 | 0.3 ± 0.03 | 0.5 ± 0.1 |
| PID 29 | 3.3 ± 0.5 | 8.5 ± 1.2 | 0.98 ± 0.4 | 32.7 ± 3.3 | 0.9 ± 0.5 | 3.8 ± 1.2 | 0.99 ± 0.4 | 0.4 ± 0.2 |
| PID 40 | 3.0 ± 0.9 | 10.2 ± 2.3 | 1.3 ± 0.6 | 37.2 ± 7.0 | 2.0 ± 2.0 | 3.6 ± 1.6 | 1.2 ± 0.5 | 0.5 ± 0.3 |
| **MOC1 Male** | | | | | | | | |
| PID 20 | 11.3 ± 2.4 | 8.6 ± 0.2 | 7.8 ± 1.7 | 36.6 ± 3.9 | 3.5 ± 0.1 | 3.3 ± 1.3 | 0.1 ± 0.01 | 2.2 ± 0.5 |
| PID 29 | 9.8 ± 1.1 | 9.6 ± 1.3 | 18.5 ± 2.3 | 28.0 ± 5.9 | 4.5 ± 1.0 | 5.8 ± 4.0 | 0.39 ± 0.2 | 1.8 ± 1.2 |
| PID 40 | 9.0 ± 0.7 | 7.6 ± 2.4 | 30.4 ± 15.6 | 20.7 ± 7.5 | 4.3 ± 1.5 | 2.3 ± 0.8 | 0.4 ± 0.1 | 1.6 ± 0.7 |
| **MOC1 Female** | | | | | | | | |
| PID 20 | 13.2 ± 4.4 | 8.7 ± 0.7 | 7.3 ± 0.8 | 32.7 ± 3.0 | 3.4 ± 0.2 | 4.9 ± 1.6 | 0.1 ± 0.1 | 1.1 ± 0.2 |
| PID 29 | 7.8 ± 1.0 | 9.4 ± 0.9 | 13.0 ± 3.2 | 32.7 ± 1.2 | 3.8 ± 0.7 | 4.3 ± 0.6 | 0.3 ± 0.04 | 0.5 ± 0.2 |
| PID 40 | 9.4 ± 0.9 | 9.2 ± 2.2 | 6.7 ± 0.6 | 28.5 ± 5.1 | 5.6 ± 0.4 | 2.8 ± 0.7 | 0.2 ± 0.1 | 1.9 ± 0.4 |
| **Sham Male** | | | | | | | | |
| PID 6 | 4.7 ± 0.5 | 8.2 ± 1.8 | 2.7 ± 2.3 | 34.7 ± 0.6 | 0.6 ± 0.3 | 1.9 ± 0.2 | 0.7 ± 0.1 | 0.6 ± 0.3 |
| PID 9 | 26.7 ± 4.7 | 2.7 ± 1.4 | 35.1 ± 11.4 | 35.5 ± 7.9 | 1.2 ± 0.3 | 0.2 ± 0.03 | 0.2 ± 0.03 | 0.6 ± 0.1 |
| PID 12 | 4.0 ± 0.7 | 8.2 ± 1.9 | 16.6 ± 4.5 | 36.4 ± 2.6 | 1.1 ± 0.7 | 0.9 ± 0.2 | 0.9 ± 0.3 | 0.1 ± 0.03 |
| **Sham Female** | | | | | | | | |
| PID 6 | 5.0 ± 0.4 | 7.2 ± 0.9 | 2.8 ± 1.5 | 36.7 ± 0.5 | 0.5 ± 0.1 | 0.9 ± 0.2 | 0.7 ± 0.1 | 0.3 ± 0.2 |
| PID 9 | 4.2 ± 0.4 | 11.2 ± 2.0 | 2.6 ± 1.2 | 42.4 ± 1.9 | 0.6 ± 0.3 | 1.0 ± 0.3 | 0.4 ± 0.1 | 0.6 ± 0.2 |
| PID 12 | 3.7 ± 0.7 | 7.0 ± 6.9 | 1.9 ± 1.7 | 33.6 ± 5.4 | 0.4 ± 0.2 | 1.1 ± 0.3 | 0.6 ± 0.3 | 0.1 ± 0.05 |
| **MOC2 Male** | | | | | | | | |
| PID 6 | 17.4 ± 3.9 | 3.2 ± 0.3 | 30.4 ± 5.0 | 40.4 ± 2.9 | 1.1 ± 0.1 | 0.1 ± 0.04 | 0.5 ± 0.2 | 0.3 ± 0.04 |
| PID 9 | 26.7 ± 4.7 | 2.7 ± 1.4 | 35.1 ± 11.4 | 35.5 ± 7.9 | 1.2 ± 0.3 | 0.2 ± 0.03 | 0.2 ± 0.03 | 0.6 ± 0.1 |
| PID 12 | 16.9 ± 2.3 | 3.2 ± 1.7 | 35.6 ± 11.0 | 32.8 ± 6.5 | 1.1 ± 0.4 | 0.3 ± 0.1 | 0.3 ± 0.1 | 0.2 ± 0.1 |
| **MOC2 Female** | | | | | | | | |
| PID 6 | 21.0 ± 2.1 | 3.0 ±1.0 | 29.2 ± 6.8 | 39.5 ± 6.8 | 0.9 ± 0.03 | 0.1 ± 0.03 | 0.2 ± 0.04 | 0.4 ± 0.2 |
| PID 9 | 203 ± 2.5 | 2.6 ± 0.4 | 29.8 ± 3.4 | 38.2 ± 1.6 | 1.0 ± 0.04 | 0.2 ± 0.02 | 0.2 ± 0.1 | 0.8 ± 0.2 |
| PID 12 | 20.1 ± 7.4 | 1.4 ± 0.4 | 52.2 ± 6.8 | 22 ± 4.8 | 0.8 ± 0.12 | 0.2 ± 0.02 | 0.3 ± 0.2 | 0.1 ± 0.03 |
