# Supplementary material for: The impact of tumor immunogenicity on cancer pain phenotype using syngeneic oral cancer mouse models
Source: Front Pain Res (Lausanne). 2022 Sep 12;3:991725. doi: 10.3389/fpain.2022.991725 (PMC9512086; doi:10.3389/fpain.2022.991725)
Supplement: Supplementary file 2 [file Table_2.DOCX]

**Supplemental Table 2: Immune cell subtypes in the draining lymph tissue**

| **Sample** | ***CD45^+^** | **Myeloid Derived** | | | **Lymphocyte** | | | |
| --- | --- | --- | --- | --- | --- | --- | --- | --- |
|  |  | **Cd11c^+^** | **Ly6g^+^** | **F4/80^+^** | **CD4^+^** | **CD8^+^** | **CD19^+^** | **NK1.1^+^** |
| **Sham Male** | | | | | | | | |
| PID 20 | 59.8 ± 8.7 | 0.3 ± 0.03 | 0.1 ± 0.03 | 1.3 ± 0.3 | 21.9 ± 2.9 | 9.3 ± 2.2 | 46.7 ± 4.6 | 0.6 ± 0.1 |
| PID 29 | 54.7 ± 18.3 | 0.3 ± 0.07 | 0.08 ±0.04 | 1.7 ± 0.4 | 21.2 ± 3.9 | 9.9 ± 2.6 | 46.2 ± 6.5 | 0.4 ± 0.06 |
| PID 40 | 54.9 ± 3.6 | 0.3 ± 0.01 | 0.1 ± 0.02 | 1.5 ± 0.2 | 20.5 ± 2.2 | 9.5 ± 0.4 | 48.5 ± 3.6 | 0.5 ± 0.1 |
| **Sham Female** | | | | | | | | |
| PID 20 | 51.1 ± 18.3 | 0.2 ± 0.03 | 0.1 ± 0.03 | 1.3 ± 0.1 | 23.7 ± 2.8 | 12.9 ± 2.3 | 42.7 ± 0.7 | 0.5 ± 0.1 |
| PID 29 | 64.0 ± 9.7 | 0.3 ± 0.03 | 0.1 ± 0.05 | 1.4 ± 0.4 | 23.3 ± 0.9 | 11.9 ± 2.5 | 44.4 ± 1.8 | 0.5 ± 0.07 |
| PID 40 | 58.1 ± 4.4 | 0.4 ± 0.08 | 0.2 ± 0.06 | 1.9 ± 0.5 | 24.2 ± 3.3 | 11.8 ± 1.6 | 42.2 ± 5.0 | 0.5 ± 0.1 |
| **MOC1 Male** | | | | | | | | |
| PID 20 | 84.9 ± 4.3 | 0.3 ± 0.02 | 0.1 ± 0.08 | 1.5 ± 0.3 | 16.2 ± 1.9 | 7.9 ± 0.6 | 61.2 ± 3.5 | 0.5 ± 0.1 |
| PID 29 | 77.3 ± 103 | 0.3 ± 0.05 | 0.09 ± 0.02 | 1.4 ± 0.2 | 17.9 ± 2.3 | 8.2 ± 0.7 | 57.6 ± 6.0 | 0.4 ± 0.09 |
| PID 40 | 85.0 ± 5.4 | 0.3 ± 0.1 | 0.5 ± 0.3 | 1.6 ± 0.3 | 13.9 ± 1.7 | 6.4 ± 0.6 | 67.5 ± 2.8 | 0.4 ± 0.1 |
| **MOC1 Female** | | | | | | | | |
| PID 20 | 87.1 ± 1.7 | 0.4 ± 0.04 | 0.1 ± 0.03 | 1.7 ± 0.3 | 20.0 ± 1.7 | 11.6 ± 1.5 | 50.3 ± 4.8 | 0.5 ± 0.07 |
| PID 29 | 87.7 ± 3.7 | 0.4 ± 0.04 | 1.7 ± 0.04 | 1.6 ± 0.1 | 21.0 ± 2.9 | 10.9 ± 2.4 | 50.8 ± 3.2 | 0.4 ± 0.04 |
| PID 40 | 85.8 ± 3.6 | 0.4 ± 0.1 | 0.3 ± 0.3 | 1.9 ± 0.6 | 15.4 ± 1.3 | 8.7 ± 2.0 | 65.2 ± 2.9 | 0.4 ± 0.02 |
| **Sham Male** | | | | | | | | |
| PID 6 | 70.0 ± 7.5 | 0.3 ± 0.0 | 0.1 ± 0.0 | 1.6 ± 0.3 | 21.4 ± 1.4 | 8.7 ± 1.2 | 51.6 ± 4.5 | 0.4 ± 0.0 |
| PID 9 | 53.7 ± 5.1 | 0.2 ± 0.1 | 0.2 ± 0.0 | 1.9 ± 0.5 | 8.8 ± 5.0 | 3.7 ± 2.6 | 42.2 ± 5.3 | 0.5 ± 0.0 |
| PID 12 | 63.3 ± 1.6 | 0.3 ± 0.0 | 0.2 ± 0.0 | 1.8 ± 0.3 | 18.8 ± 3.0 | 8.9 ± 1.3 | 49.0 ± 1.9 | 0.5 ± 0.1 |
| **Sham Female** | | | | | | | | |
| PID 6 | 69.6 ± 9.7 | 0.3 ± 0.1 | 0.1 ± 0.0 | 1.1 ± 0.2 | 22.2 ± 3.9 | 10.5 ± 1.1 | 49.8 ± 5.0 | 0.4 ± 0.0 |
| PID 9 | 39.9 ± 2.5 | 0.3 ± 0.2 | 0.2 ± 0.1 | 1.8 ± 0.5 | 15.3 ± 8.5 | 4.2 ± 3.6 | 34.8 ± 9.0 | 0.5 ± 0.1 |
| PID 12 | 76.0 ± 0.9 | 0.4 ± 0.1 | 0.2 ± 0.1 | 1.9 ± 0.4 | 23.5 ± 2.8 | 12.1 ± 2.7 | 43.7 ± 5.6 | 0.5 ± 0.1 |
| **MOC2 Male** | | | | | | | | |
| PID 6 | 84.9 ± 8.7 | 0.4 ± 0.1 | 0.3 ± 0.1 | 2.4 ± 0.8 | 13.4 ± 0.9 | 4.6 ± 1.0 | 68.5 ± 3.2 | 0.4 ± 0.1 |
| PID 9 | 90.2 ± 2.5 | 0.3 ± 0.1 | 0.9 ± 0.5 | 3.5 ± 1.3 | 6.4 ± 2.6 | 2.1 ± 0.9 | 70.3 ± 3.1 | 0.6 ± 0.2 |
| PID 12 | 80.4 ± 9.4 | 0.3 ± 0.1 | 1.3 ± 1.2 | 4.0 ± 0.2 | 11.1 ± 2.0 | 5.8 ± 0.9 | 67.1 ± 3.7 | 0.4 ± 0.1 |
| **MOC2 Female** | | | | | | | | |
| PID 6 | 82.6 ± 2.6 | 0.5 ± 0.1 | 0.5 ± 0.1 | 3.1 ± 0.1 | 14.6 ± 1.0 | 6.0 ± 0.7 | 65.3 ± 2.1 | 0.5 ± 0.1 |
| PID 9 | 85.0 ± 9.3 | 0.3 ± 0.1 | 0.5 ± 0.0 | 3.3 ± 0.5 | 11.2 ± 4.5 | 4.8 ± 2.5 | 60.5 ± 5.2 | 0.6 ± 0.1 |
| PID 12 | 78.5 ± 9.6 | 0.2 ± 0.0 | 1.0 ± 0.4 | 3.3 ± 0.7 | 12.1 ± 1.1 | 6.5 ± 1.4 | 66.4 ± 3.2 | 0.3 ± 0.1 |
